# Microstructure and Composition of Full Fat Cheddar Cheese Made with Ultrafiltered Milk Retentate

**DOI:** 10.3390/foods2030310

**Published:** 2013-07-18

**Authors:** Lydia Ong, Raymond R. Dagastine, Sandra E. Kentish, Sally L. Gras

**Affiliations:** 1 Particulate Fluid Processing Centre, Department of Chemical and Biomolecular Engineering, The University of Melbourne, Parkville, VIC 3010, Australia; E-Mails: lon@unimelb.edu.au (L.O.); rrd@unimelb.edu.au (R.R.D.); sandraek@unimelb.edu.au (S.E.K.); 2 The Bio21 Molecular Science and Biotechnology Institute, The University of Melbourne, Parkville, VIC 3010, Australia

**Keywords:** Cheddar cheese, fat retention, microstructure, ultrafiltration

## Abstract

Milk protein is often standardised prior to cheese-making using low concentration factor ultrafiltration retentate (LCUFR) but the effect of LCUFR addition on the microstructure of full fat gel, curd and Cheddar cheese is not known. In this work, Cheddar cheeses were made from cheese-milk with or without LCUFR addition using a protein concentration of 3.7%–5.8% w/w. The fat lost to sweet whey was higher in cheese made from cheese-milk without LCUFR or from cheese-milk with 5.8% w/w protein. At 5.8% w/w protein concentration, the porosity of the gel increased significantly and the fat globules within the gel and curd tended to pool together, which possibly contributed to the higher fat loss in the sweet whey. The microstructure of cheese from cheese-milk with a higher protein concentration was more compact, consistent with the increased hardness, although the cohesiveness was lower. These results highlight the potential use of LCUFR for the standardization of protein concentration in cheese-milk to 4%–5% w/w (equivalent to a casein to total protein ratio of 77%–79% w/w) to increase yield. Beyond this concentration, significant changes in the gel microstructure, cheese texture and fat loss were observed.

## 1. Introduction

The protein concentration of milk varies according to the season, weather, feed type, stage of lactation and breed of lactating cows. This variation has a major influence on the coagulation of milk and production of cheese. Milk standardization minimizes the effect of the variations in milk on the composition and quality of cheese. Several studies have reported the use of milk ultrafiltration (UF) retentate for milk standardization prior to cheese-making, highlighting the potential benefits of standardization with UF, including an increase in cheese yield and plant productivity [1,2,3,4,5,6,7]. Higher milk protein concentrations also result in less whey being expelled during cheese making, reducing the volume of whey that requires processing [3].

The UF preparation used most widely in the standardization of cheese-milk for Cheddar cheese manufacture is low concentration UF retentate (LCUFR), with a concentration factor of approximately 1.6–1.7 fold [8]. The process of ultrafiltration changes the milk composition, increasing the total solids content and decreasing the non-protein nitrogen (NPN), soluble calcium and lactose, as these latter components permeate through the UF membrane. Ultrafiltration can also cause a decrease in micelle size and number of hydrophobic sites on the surface of milk proteins, as assessed by 1-anilinonaphthalene-8-sulfonate (ANS) binding [9]. These surface changes in turn further decrease the surface potential of casein micelles.

The changes in the milk properties after ultrafiltration are known to affect rennet induced coagulation [10,11,12,13] and the rheological properties of rennet gels have been comprehensively examined. Studies on the effect of LCUFR on the microstructure of the gel, curd and cheese are, however, limited. 

In a previous study where skim milk retentate was used to give 3%–19% w/w protein, the gels formed from UF concentrated milk were much firmer, as the increased milk protein concentration decreased the mean distance between casein micelles [10]. This proximity increased the rate of aggregation of para-casein micelles, as aggregation depends on the number of effective collisions. A study by Green [7] looked at the microstructure of the gel, curd and cheese progressively during Cheddar cheese making from skim milk that was concentrated 1.7–4 fold and then blended with cream. The samples were examined by a conventional scanning electron microscopy (SEM) and transmission electron microscopy (TEM), however, only the microstructure of the protein was shown as fat is removed during sample preparation for conventional SEM or TEM [7]. Green found that the 2–4 fold UF concentration lead to a coarser protein network, a reduced volume of whey and more fat in the whey compared to cheese made using unconcentrated milk. Curd formation was faster when a higher concentration of milk protein was used, despite the reduced ratio of rennet to protein and the final texture of the cheese tended to be granular and hard. The authors used light microscopy to examine the distribution of the fat globules but only details of the surface microstructure could be obtained using this method [7]. 

Other microstructure studies have only focused on the very early stages of cheese making. Confocal laser scanning microscopy (CLSM), for example, was used to look at the microstructure of the gel from UF concentrated skim milk with a casein content enriched from 2.75% to 19.8% w/w [14]. The UF gel contained smaller aggregates and fewer hydrolysed kappa casein molecules as compared to in unconcentrated milk (20% c.f. 50%). After 24 h at 30 °C, syneresis was observed and the protein strands within the concentrated samples were longer and thicker. Although this study provides useful insight into the structure of gels made using UF concentrated skim milk, no cheese making was performed to examine the effect of the altered gel structure on the microstructure of curd and final cheese.

The present study looks beyond the changes in the microstructure of the gel to later processing steps. Curd and cheese are prepared using pilot scale manufacturing conditions with cheese-milk and UF retentate from an industrial scale process; so that results are highly applicable to commercial production. The present study also applies advanced microscopy techniques including cryo SEM and CLSM to investigate the microstructure of samples made using cheese-milk standardized with LCUFR. The ability to preserve the fat within the curd microstructure under cryogenic conditions provides new insights into the distribution and changes to the fat microstructure during manufacturing. The influence of microstructure on the composition, texture and yield of the final cheese product is also examined.

## 2. Experimental Section

### 2.1. Acidification of Cheese-Milk by Starter Bacteria

The effect of protein concentrations of 4%, 5% or 6% w/w on the acidification of milk by starter bacteria was investigated prior to cheese making experiments. Target protein concentrations were achieved by blending raw milk with raw low concentration factor ultrafiltration retentate (LCUFR, protein = 7.46% w/w and fat = 8.62% w/w) obtained from an ultrafiltration process at 14 °C. Cream was then added to the milk to achieve a final fat to protein ratio of 0.84 before pasteurization at 72 °C for 15 s. All milk preparations were obtained from a local cheese factory (Murray Goulburn, Melbourne, VIC, Australia). Freeze dried mixed strain direct vat set (DVS) mesophilic starter culture (0.13 U/kg; Chr. Hansen, Melbourne, VIC, Australia) was then added to the pasteurized and standardized cheese-milk at 33 °C and incubated for 330 min. The pH of the milk was recorded at 15 min intervals in the first 150 min then at 60 min intervals until the end of the fermentation. The experiment was repeated 3 times (*n* = 3).

### 2.2. Manufacture of Cheddar Cheese

Four batches of Cheddar cheeses were made in triplicate at the Murray Goulburn pilot plant, Cobram, VIC, Australia. The experimental plan is shown in Table 1. Batch 1 was made using pasteurized raw milk with no protein standardization (the fat was standardized as described in Section 2.1). Batches 2–4 were made using milk prepared with a target protein concentration of 4%, 5% and 6% w/w respectively. The protein and fat concentration of the milk was also achieved using the standardization method described in Section 2.1. These milk preparations were then used for cheese making and are referred to as cheese-milk.

**Table 1:** Experimental plan for pilot scale trial investigating the effect of protein concentration on the microstructure and composition of Cheddar cheese.

Batch	Target milk protein (% w/w)	Weight of milk in cheese vat (kg)	Milk fat (% w/w) ^#^	Milk protein (% w/w) ^#^	Starter culture concentration (U/kg milk) *	Starter culture concentration in relation to milk protein content (U/kg protein) *	Ripening time ^ (min)	Rennet concentration (mL/kg milk)	Rennet concentration in relation to milk protein content (mL/kg protein)^ #^
1	3.5	235	4.37 ± 0.06	3.69 ± 0.05	0.11	3.0 ± 0.2	20	0.06	1.7 ± 0.1
2	4.0	200	4.83 ± 0.02	4.02 ± 0.01	0.13	3.1 ± 0.0	20	0.06	1.5 ± 0.0
3	5.0	160	5.67 ± 0.61	4.79 ± 0.34	0.16	3.3 ± 0.2	30	0.06	1.2 ± 0.1
4	6.0	135	6.97 ± 0.10	5.76 ± 0.06	0.19	3.2 ± 0.0	40	0.06	1.0 ± 0.0

* 10 U of freeze dried culture corresponds to 1 L of active bulk culture, defined here as active starter culture ready to be used for cheese-making; ^ Ripening time is the time from starter addition to rennet addition; ^#^ Results are expressed as mean ± standard deviation of mean (*n* = 3).

The total protein in each cheese vat was normalised to approximately 8 kg of protein by decreasing the volume of milk used for preparations with an increased protein content (Table 1). This ensured that the curds prepared using preparations with a higher protein content could fit within the 12 kg limitation of the cheese press. Four cheese-presses were available and two batches of cheese were made each day giving 4 blocks of cheese each 10–12 kg in weight. After pasteurization, the cheese-milk was cooled to 33 °C before inoculation with 25 U/vat starter culture (Chr. Hansen, Melbourne, VIC, Australia) to give final starter culture concentration as shown in Table 1. The milk was then ripened for 20 to 40 min until the pH of the milk reached approximately pH 6.5 as shown in Table 1. Rennet (Hannilase, 690 IMCU/mL; Chr. Hansen, Melbourne, VIC, Australia) was added to give a final concentration of 0.06 mL/kg of milk for all treatments. The milk was allowed to coagulate at 33 °C for 20–45 min as determined in Section 2.3. The consistency of the gel made using the different protein concentrations before cutting was classified as “medium set” by an experienced cheese maker. The gel was cut over a period of 20 min and cooked from 33 °C to 38 °C with stirring over a period of 45 min. The sample was then cooked at 38 °C with stirring until the pH of the samples reached approximately pH 6.2. The sweet whey was released and collected for compositional analysis.

Cooked curd samples were piled into blocks ~15 cm in height and turned every 15 min during cheddaring until the pH of the curd reached pH 5.3. Curd was milled and salted with 770 g salt per vat before pressing at 200 psi. The salty whey that was released during pressing was collected and the composition analysed.

### 2.3. Viscoelastic Properties of Cheese-Milk with Different Protein Concentrations

The storage modulus (G’) of cheese-milk with different protein concentrations were analysed before the main cheese-making experiments using a rheometer (ARES-TA Instruments, New Castle, DE, USA), as described previously [15]. A dynamic time sweep analysis at angular frequency of 0.8 Hz and 0.1% strain was used to analyze the changes in the storage modulus (G’) as the milk gelled. The time taken for the cheese-milk to first reach a G’ of 60 Pa was recorded and used as a guide for the cutting time in the pilot scale cheese-making experiments. The experiment was performed twice (*n* = 2).

### 2.4. CLSM, Cryo SEM and Image Analysis

The microstructure of the gel, curd and cheese samples were analysed using a Confocal Laser Scanning Microscopy (Leica Microsystems, Heidelberg, Germany) and a cryo Scanning Electron Microscopy (Quanta; Fei Company, Hillsboro, OR, USA) using the method reported in a previous study [16]. 

The cooked curd samples were collected from the cheese vat immediately after the whey draining process. Milled curd was collected prior to salting. The cheese samples were collected after 16 h of pressing. Samples were sealed in air tight containers to prevent drying and kept at 4 °C for analysis within 3 days of sample collection. Gel samples were prepared separately in the laboratory using the same cheese-milk used for the pilot scale cheese-making trial and observed immediately within 15 min after gel formation. The details of sample preparation for CLSM and cryo SEM observations have been reported in a previous study [16]. 

The 3D CLSM image reconstruction and analysis were performed using a commercial software package (Imaris, Bitplane, South Windsor, CT, USA), as described previously [15]. Quantitative outputs such as the number of fat globules per unit volume, their sphericity, mean volume and diameter, the total volume of fat and the fraction of pore volume with respect to the total sample volume (porosity) were obtained from the image analysis. Two 3D images were taken for each batch of the cheese produced. Three batches of cheeses were produced for each milk protein concentration, giving a total of 6 analyses for each data point presented from image analysis. 

### 2.5. Compositional Analysis, Fat and Protein Retention and Cheese Yield

A Milko Scan FT120 (FOSS, North Ryde, NSW, Australia) was used to analyse the protein and fat content of the milk and sweet whey. Non-casein nitrogen (NCN) and non protein nitrogen (NPN) within the milk samples were determined using Kjeldahl analysis from the filtrate obtained after selective precipitation at pH 4.6 or the filtrate from a 12% trichloroacetic acid solution, as described in AOAC Methods 991.20 and 991.21 [17]. The casein content was calculated by difference from the NCN concentration and total protein concentration. 

The fat, protein, total solids, salt, pH and moisture content of the cheese were analysed as described previously [15]. The fat and protein lost in the whey or retained in the cheese, the cheese yield and dry matter cheese yield were also calculated as described in Ong *et al.* [15]. The total amount of protein and fat lost in the whey plus that retained in the cheese was in all cases between 93% and 99% of the initial amount of each ingredient added.

### 2.6. Texture Analysis

Texture Profile Analysis was performed on cheese samples using a texture analyser TAXT (Stable Micro System, Surrey, UK) as described previously [15]. Texture analyses were performed six times for six independent samples from each batch of cheese giving six pseudo replicates which were then averaged. Three batches of cheeses were made for each milk protein concentration and the results were expressed as the average of three means for each protein concentration. 

### 2.7. Determination of Total Calcium Concentration in Milk and Cheese

Inductively Coupled Plasma Optical Emission Spectroscopy (ICP-OES; Varian Inc., PaloAlto, CA, USA) was used to analyse the total calcium of milk and cheese. The details of these methods have been reported elsewhere [15]. Two samples were collected from each batch and three batches of cheeses were made for each milk protein concentration, giving a total of 6 analyses for each data point presented. 

### 2.8. Enumeration of Starter Bacteria

To assess the viability of the starter bacteria and the non-starter lactic acid bacteria, the cheese samples (5 g) were diluted in 45 mL of sterile 2% (w/v) trisodium citrate (Oxoid Ltd., West Heidelberg, VIC, Australia). The sample was homogenized at 10,000 g using a high speed homogeniser (Polytron, Kinematica, Lucerne, Switzerland) for 1 min to obtain a cheese slurry for the first dilution. The homogenizer was equipped with a shaft that could be detached from the rotor. This shaft was rinsed thoroughly with warm sterile distilled water to remove any residual cheese. The shaft was then sterilized with 80% (v/v) ethanol and dried between use for samples from different cheese batches.

Subsequent serial dilutions were performed in sterile 0.15% (w/v) peptone solution (Oxoid Ltd.). The starter culture was enumerated on LM17 agar (Merck, South Granville, NSW, Australia) and incubated at 30 °C under aerobic conditions for 72 h [18]. The NSLAB was enumerated on Rogosa agar (Merck) and incubated at 30 °C in an anaerobic jar (Oxoid Ltd.) with a Gas Generating Kit^®^ (Oxoid Ltd.) for 72 h [18]. The result is presented as the mean of three results obtained from three batches of cheeses from the same milk protein concentration.

### 2.9. Statistical Analysis

Data was analysed using a statistical package from Minitab (Minitab Inc., State College, PA, USA). The differences between means were determined by one way analysis of variance (ANOVA) and Tukey’s paired comparison, with a significance level of α = 0.05.

## 3. Results and Discussion

### 3.1. The Effect of Milk Protein Concentration on the Acidification by Starter Bacteria

**Figure 1:**
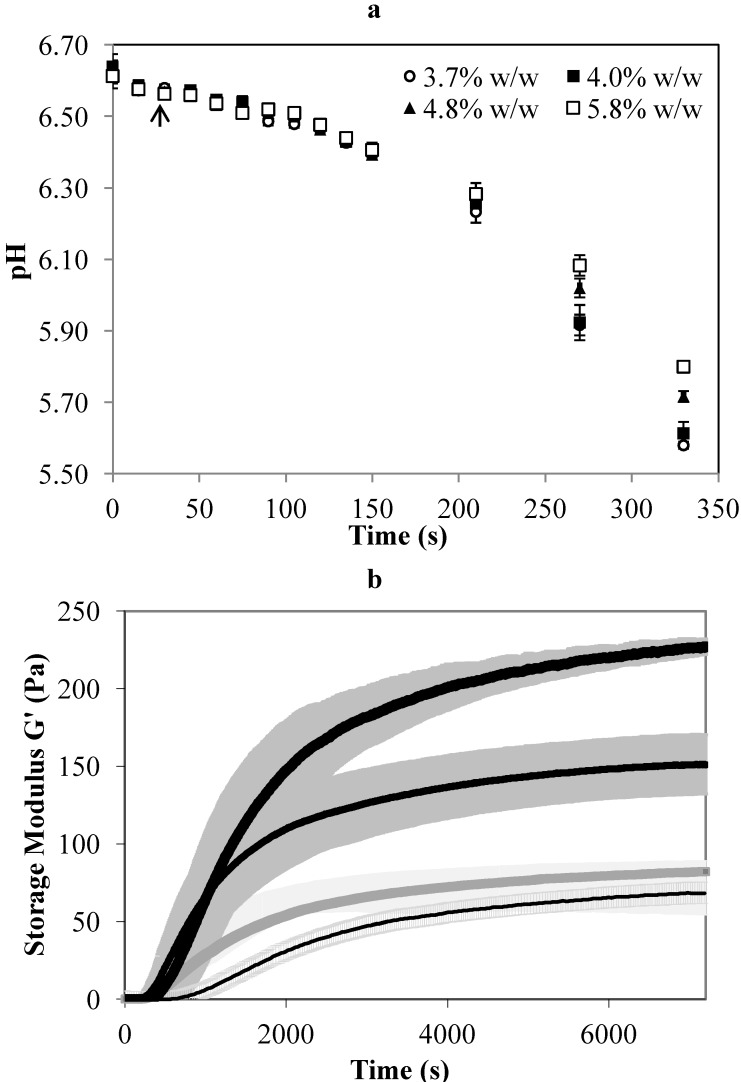
(**a**) The pH of unconcentrated cheese-milk with 3.7 % w/w milk protein and cheese-milk standardized with low concentration factor ultrafiltration retentate (LCUFR) to 4% w/w, 4.8% w/w or 5.8% w/w milk protein after starter culture addition. The arrow indicates the point where rennet is normally added. (**b**) Storage modulus (G’) measured from the time of rennet addition for unconcentrated cheese-milk with 3.7% w/w milk protein (thin line) and cheese-milk standardized with LCUFR to 4% w/w (grey line), 4.8% w/w (medium thick line) or 5.8% w/w (thick line) milk protein incubated at 33 °C. The setting time is the total time required for the sample to reach a G’ of 60 Pa. Results are expressed as mean ± standard deviation of mean from three (*n* = 3) or two (*n* = 2) independent trials for (**a**) and (**b**) respectively.

A set of laboratory experiments was conducted prior to the pilot scale cheese-making experiments, to determine the effect of milk protein concentration on the acidification and setting time of the gel. As shown in Figure 1a, the milk with a higher protein concentration (5.8% w/w protein) had a higher buffering capacity with a higher pH after incubation at 33 °C for an extended period of 330 min.

Rennet is normally added within the first 30 min of starter culture addition during Cheddar manufacture. While large variations in pH at this point may affect coagulation and the resulting microstructure, the minor differences in milk pH at the time when rennet is usually added (Figure 1a arrow) are not likely to affect the coagulation process. The starter culture concentration was increased to compensate for the increased buffering capacity so that ~3.1 U of starter was added for every kilogram of milk protein (Table 1). This adjustment prevents an increase in cooking time at the whey-off period and also ensures a similar concentration of starter culture in each cheese.

### 3.2. The Effect of Milk Protein Concentration on the Elastic Modulus and Processing Time

The storage modulus (G’) of the gel formed from milk with a higher protein concentration was significantly higher than for milk with a lower protein concentration (Figure 1b). The rate of gel firming also increased rapidly in milk with a higher protein concentration, as reflected by the gradient of the slope of the storage modulus and the decrease in the time required to obtain a given value of G’. The G’ increased rapidly between 200 and 400 s for all gels (Figure 1b), indicating a similar onset of gelation.

In our previous experiments, the time from rennet addition to cutting, known as the setting time of the gel, was set at 2700 s corresponding to a G’ of 60 Pa, as this gave a Cheddar cheese with acceptable moisture content using standardised milk with a protein concentration of 4% w/w [15]. To maintain a similar level of stiffness of 60 Pa, the setting time of the gel made with 4.8% or 5.8% w/w milk protein was set to 1200 s (Figure 1b). This adjustment prevents the curd becoming too firm and avoids curd fines in the whey. Another way to reduce the firmness of a gel is to reduce the coagulation temperature or reduce the amount of rennet used [1,19]. The concentration of rennet added per unit mass of milk was similar here (Table 1), but the rennet concentration per kg of milk protein was consequently lower in the milk with a higher protein concentration (Table 1). Further reducing the rennet for high protein treatments would impact on the residual rennet in the final cheese. For this reason, the same concentration of rennet per unit mass of milk was used in this study. The time required for the unconcentrated milk to reach G’ = 60 Pa was about 5000 s (Figure 1b). During the actual cheese-making experiment, however, the milk in the cheese vat reached a gel consistency of medium firmness and was ready for cutting at 2700 s. The longer time indicated by the rheology analysis could possibly be due to a small degree of shearing applied to the sample during analysis that disturbed the gel formation. This was not apparent in the concentrated sample, but the setting time indicated by the rheology analysis in this study was less accurate for gel made with the unconcentrated milk.

The processing time from the addition of starter culture to whey draining or milling in the pilot scale cheese-making was not significantly different (*p* > 0.05) (data not shown), suggesting that adjustments made to the starter concentration and the setting time described above were effective.

### 3.3. Composition of Cheese-Milk, Sweet Whey and Salty Whey

Ultrafiltration is known to alter the composition of milk. Approximately 81% of the protein within the LCUFR used in this study was casein as compared to 77% in unconcentrated milk. The LCUFR also contains less non protein nitrogen (NPN) arising from low molecular weight nitrogen containing compounds, such as creatine and creatinine and urea [20] as a portion of the NPN permeates through the UF membrane during ultrafiltration [21]. Consequently the concentration of NPN as a percentage of total nitrogen (TN) differed significantly in the LCUFR and unconcentrated milk from 2.5% w/w to 4.6% w/w, respectively. 

The impact of standardization of cheese-milk on casein and NPN content is shown in Table 2. As the casein concentration increased with LCUFR addition, the ratio of casein nitrogen over TN increased and the ratio of NPN over TN decreased. These different CN/TN or NPN/TN concentrations in the cheese-milk are expected to impact the final cheese yield [22].

**Table 2:** Protein composition, total solids and total calcium content of cheese-milk used for pilot scale cheese-making ^#^.

Batch	Casein (% w/w)	CN/TN (% w/w)	NPN/TN (% w/w)	Total solids (% w/w)	Total calcium (mg/kg)	P/F ratio
1	2.83 ± 0.04 ^d^	76.7 ± 0.00 ^d^	4.61 ± 0.00 ^a^	13.6 ± 0.14 ^d^	985 ± 62 ^d^	0.84 ± 0.00
2	3.12 ± 0.01 ^c^	77.5 ± 0.15 ^c^	4.19 ± 0.09 ^b^	14.4 ± 0.07 ^c^	1108 ± 31 ^c^	0.83 ± 0.01
3	3.78 ± 0.28 ^b^	78.9 ± 0.19 ^b^	3.42 ± 0.10 ^c^	15.9 ± 1.20 ^b^	1223 ± 52 ^b^	0.85 ± 0.04
4	4.60 ± 0.04 ^a^	79.9 ± 0.00 ^a^	2.83 ± 0.02 ^d^	18.1 ± 0.53 ^a^	1500 ± 34 ^a^	0.83 ± 0.00

^#^ Results are expressed as mean ± standard deviation of mean (*n* = 3); CN = casein, NPN = non protein nitrogen and TN = total nitrogen; ^A–d^ Means across a single column with different superscripts are significantly different (*p* < 0.05); Means across a single column without superscripts are not significantly different (*p >* 0.05).

**Figure 2:**
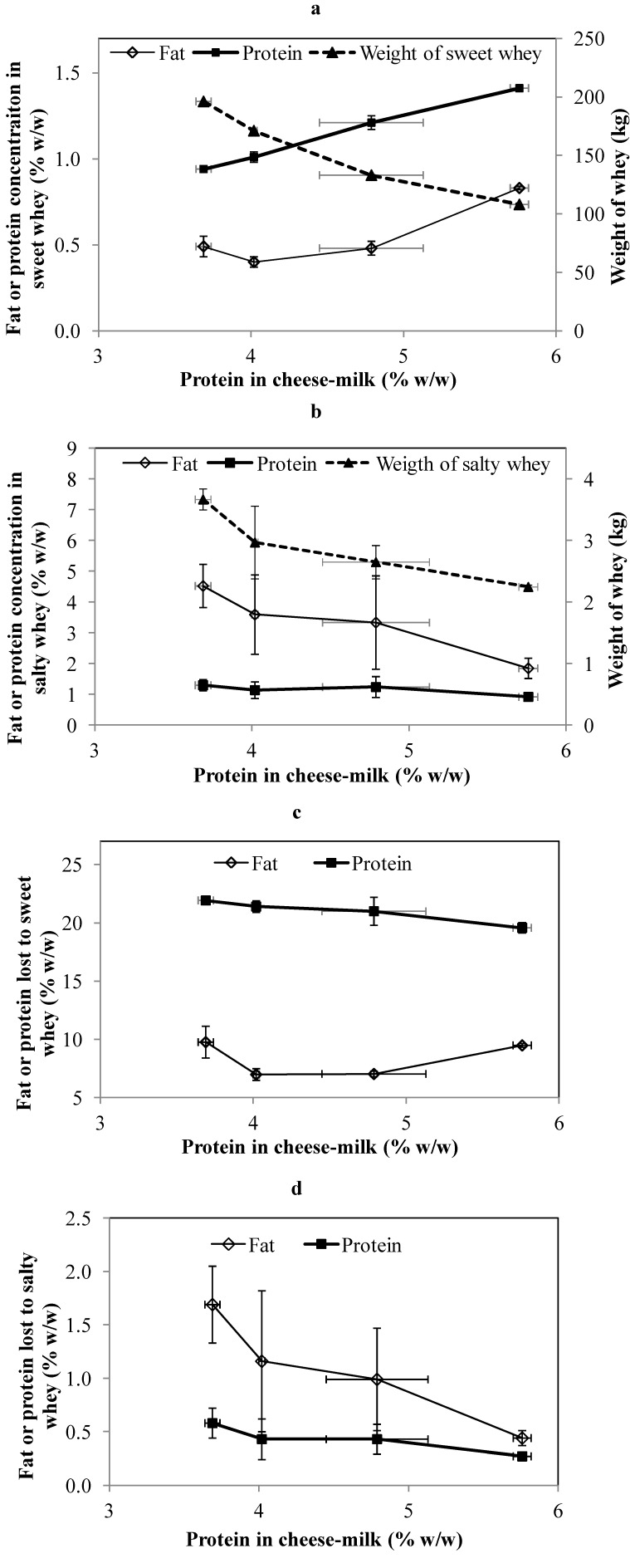
The composition and weight of sweet whey (**a**) and salty whey (**b**) collected during cheese-making at pilot scale using cheese-milk with different protein concentrations. The percentage of fat lost or protein lost to the sweet whey (**c**) and salty whey (**d**) were calculated on the basis of total fat or protein in the cheese-milk. Results are expressed as mean ± standard deviation of mean (*n* = 3).

The weight and composition of the whey generated during cheese-making at pilot scale is shown in Figure 2a,b. The weight of sweet whey and salty whey was significantly reduced (*p* < 0.05) for samples made with higher protein milk. This was expected and is consistent with previous reports that less processing of whey is required when milk with a higher protein concentration is used for cheese-making [2]. The percentage of fat lost to the sweet whey was significantly higher (*p* < 0.05) in cheese made from milk without the addition of LCUFR (Figure 2c). Greater fat loss also occurred when the concentration of milk protein was the highest (5.8% w/w) despite the increase in CN/TN, with minimum levels of fat loss occurring for samples made using cheese-milk with 4% or 4.8% (w/w) protein (Figure 2c). Conversely, while the percentage of protein in the sweet whey increased continuously as the protein in the cheese milk increased (Figure 2a), the total protein lost to this whey stream reduced continuously as the protein concentration in the cheese milk increased (Figure 2c).

The variability in fat composition measured for the salty whey was quite high. This could be due to the sampling technique used to obtain the salty whey at a pilot scale, as it was noted that some fat adhered to the cheese-press instead of flowing out with the salty whey, leading to greater variability. There is still clear evidence, however, of both a decline in the concentration of fat in the salty whey and the total fat loss (Figure 2b,d). The trends in the protein data are less clear but there is still some evidence of a decrease in protein loss into the salty whey as the protein content of the original cheese milk increases (Figure 2d). 

### 3.4. Fat and Protein Retention, the Composition and Yield of Cheese

**Figure 3:**
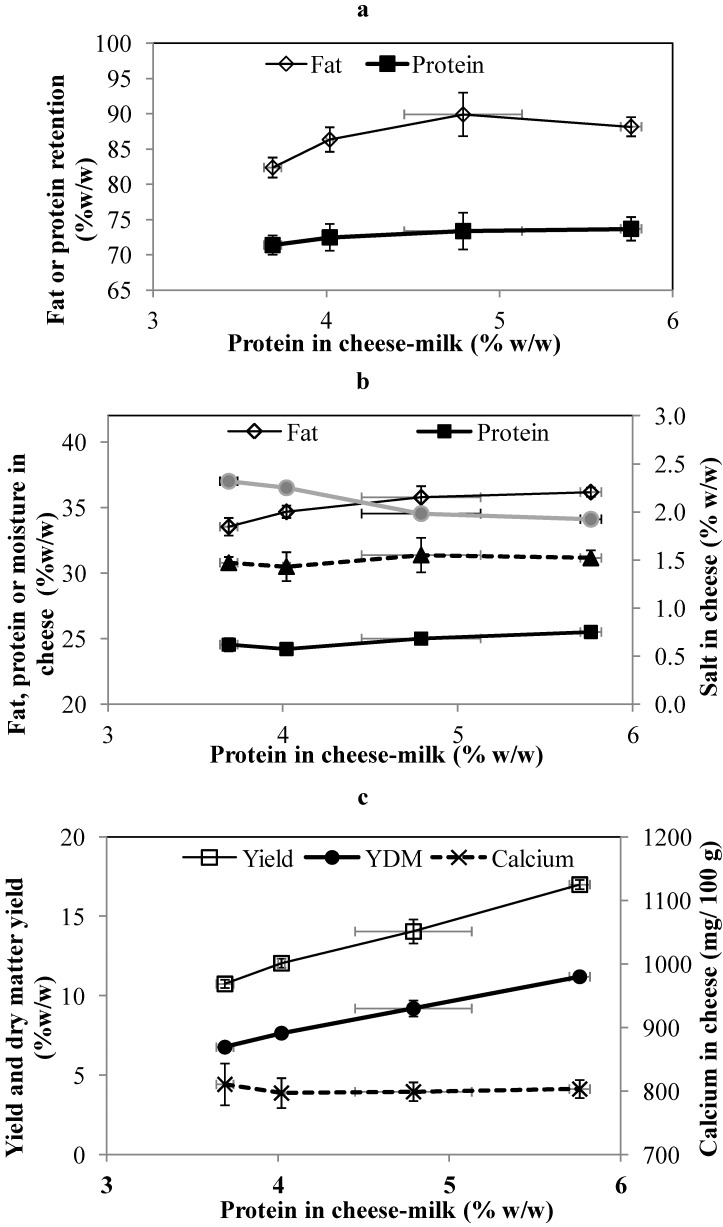
The fat and protein retention (**a**), composition (**b**) and yield (**c**) of cheese prepared at pilot scale using cheese-milk with different protein concentrations. Fat retention or protein retention in the cheese were calculated on the basis of total fat or protein in the cheese-milk. Results are expressed as mean ± standard deviation of mean (*n* = 3).

Consistent with the changes in the sweet and salty whey, the percentage of protein retained in the cheese increased with the protein in the cheese milk, although the trend is within experimental error (Figure 3a). The percentage of fat retained in the cheese also increases up to a level of 4.8% w/w total protein in the cheese milk. No further increase is observed at 5.8 w/w protein. In a similar study, Govindasamy-Lucey *et al.* [3] reported no significant difference in fat retention when increasing the milk protein concentration from 3.2% to 5.0% w/w using whole milk UF retentate [3]. Overall, the mean values of fat retention for all cheeses (Figure 3a) were within the range of values (~83%–92% w/w) reported elsewhere for full fat Cheddar cheese [23].

The composition of all cheeses was also within the acceptable range for Cheddar cheese. The fat content of the cheese made with LCUFR standardized cheese-milk with 4.8% or 5.8% w/w protein was significantly higher (*p* < 0.05) than cheese made using unconcentrated cheese-milk (Figure 3b). This could be due to the higher fat retention but also the lower moisture content of these cheeses (Figure 3b). The effect on cheese moisture suggests that the addition of LCUFR could be used as a tool to reduce the moisture content of Cheddar cheese when seasonal differences make it difficult to achieve a low moisture Cheddar [24]. The protein and salt content were not significantly different between different treatments (*p* > 0.05, Figure 3b). Our results are in agreement with a previous study [1], where milk protein level was increased in the range of 30–70 g/L with little effect on Cheddar composition. 

The weight of the cheeses made using cheese-milk with ~3.7%, 4%, 4.8% and 5.8% w/w protein were 24.4 ± 1.7, 24.1 ± 0.56, 22.5 ± 1.3 and 23.0 ± 0.41 kg respectively. The yield calculation on the basis of milk volume and the dry matter yield increased linearly with protein content (Figure 3c), due to the higher protein and total solids content of the LCUFR standardized cheese-milk (Table 2).

The concentration of total calcium in the cheese-milk increased with the increased concentration of protein (Table 2), although the ratio of total calcium (TC) over total protein (TP) in the cheese-milk was similar at 24 mg/g to 28 mg/g (*p* > 0.05). The concentration of TC in the whey was not significantly different (*p* > 0.05). The TC in the whey was 380 ± 32, 392 ± 40, 383 ± 26 and 416 ± 29 mg/kg from cheese-making with 3.7%, 4%, 4.8% and 5.8 % w/w milk protein, respectively. Increasing the concentration of milk protein did not affect the total calcium in the final cheese (Figure 3c). Less milk was added to the cheese vat for the treatments with increasing concentrations of protein. This adjustment led to similar amount of TC at a range between 230 g to 200 g in all vats regardless of protein concentration. 

### 3.5. Microstructure of Gel, Curd and Cheese

**Figure 4:**
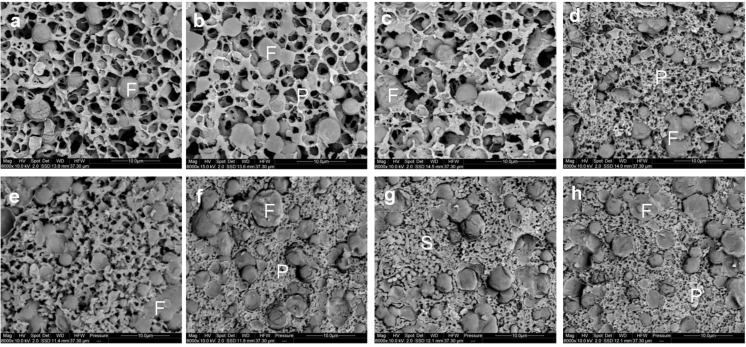
Cryo SEM micrographs of gels (**a**–**d**) and cooked curd (**e**–**h**) made using (**a**,**e**) unconcentrated cheese-milk with ~3.7% w/w protein or cheese-milk standardized with LCUFR to (**b**,**f**) 4% w/w, (**c**,**g**) 4.8% w/w or (**d**,**h**) 5.8% w/w protein, where F = fat, P = protein network and S= starter culture bacteria. The gel samples were fixed in slushed liquid nitrogen when it reached the cutting time at 60 Pa. Scale bars within the images are 10 μm in length.

The microstructure of the gel observed using cryo SEM is shown in Figure 4a–d. After the addition of rennet, casein micelles aggregate to form a protein network in which the fat globules are entrapped. Qualitatively, there was no clear distinction between the microstructure of the gels made using cheese-milk with ~3.7%–4.8% w/w milk protein (Figure 4a–c respectively). The microstructure of the gel with 5.8% w/w milk protein, however, was denser with smaller pores (indicated by the black areas in Figure 4d). This dense network might arise due to the increased aggregation of casein micelles. As discussed previously, the high concentration of protein in the samples decreased the mean free distance between casein micelles, resulting in a densely aggregated CM network. 

It is interesting to note that the gel stiffness was similar for all samples regardless of the protein concentration during cutting but the structure of the gel made from milk with 5.8% protein was very different. The stiffness of the gel could be attributed to two factors; the number of aggregating CM and the strength of the bonds between the CM and the relative importance of these factors may differ between treatments. 

**Figure 5:**
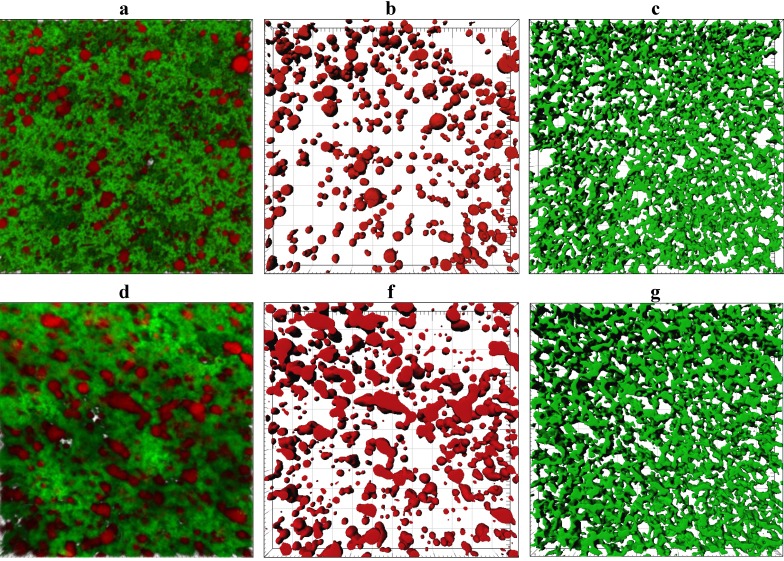
Confocal laser scanning microscopy (CLSM) microstructure of gels made using unconcentrated cheese-milk with ~3.7% w/w milk protein (**a**–**c**) or cheese-milk standardized with LCUFR to 5.8% w/w milk protein (**d**–**f**). All images are 3D reconstructions consisting of 40 layers where the separation between layers is 0.25 µm giving a total observation depth of 10 µm. Nile red stained fat appears red and fast green stained protein appears green. Images **b**–**f** are the rendered volume of the CLSM images, where **b** and **e** show the rendered volume of the fat; **c** and **f** show the rendered volume of the protein. All scale bars are 20 µm in length.

Representative CLSM images showing the microstructure of gel made from ~3.7% and 5.8% w/w milk protein are shown in Figure 5 and a complete set of 2D CLSM images is given in supplementary Figure S1. Despite a difference of ~2% in fat content between cheese-milk with ~3.7% w/w and 5.8% w/w protein samples, the number of fat globules and the total volume of the fat globules were not significantly different within the gel (*p* > 0.05, supplementary Figure S2a,e). The image analysis output shows a variation greater than 2% which made it hard to detect the small variation in fat content. The sphericity, fat droplet volume and fat droplet diameter were also similar (*p* > 0.05, supplementary Figure S2b–d). A lower porosity was observed for the gel with 5.8% w/w milk protein (supplementary Figure S2f), consistent with the denser structure observed by cryo SEM. 

CLSM provides a poorer resolution of the protein network compared to cryo SEM but a better overview of the distribution of fat and an even distribution of fat was seen within the gel made using the unconcentrated milk (Figure 5b). The porous nature of the protein network in this sample (Figure 5c), however, suggests some of this fat may be lost to the whey after cutting. In contrast, the fat globules tended to pool together at higher protein concentrations and the fat distribution was more heterogeneous (Figure 5e).

Due to the nature of the pilot study occurring at a manufacturing site, samples of cooked curd, milled curd and cheese were stored at 4 °C for 2 days prior to observation by CLSM. A separate laboratory study was conducted to assess the impact of sample storage and ensure that any small changes in structure did not cause differences between treatments. A small degree of compaction was observed for all these samples regardless of protein content (data not shown) and a small degree of syneresis observed for the cooked curd. The trend of samples made from 5.8% w/w protein displaying a denser structure was not found to be obscured by storage. 

The microstructure of the cooked curd samples produced at a pilot scale as observed by cryo SEM, is shown in Figure 4e–h. The heat treatment applied during cooking at 38 °C resulted in further fusion of CM particles within each sample compared to the gel (Figure 4a–d). Some bacterial cells could also be observed within the cooked curd sample (Figure 4g). The only difference in the observed microstructure was the bigger pores remaining within the cooked curd prepared using the unconcentrated cheese-milk (Figure 4e), as compared to cooked curd made using milk with a higher protein concentration (Figure 4f–h). 

**Figure 6:**
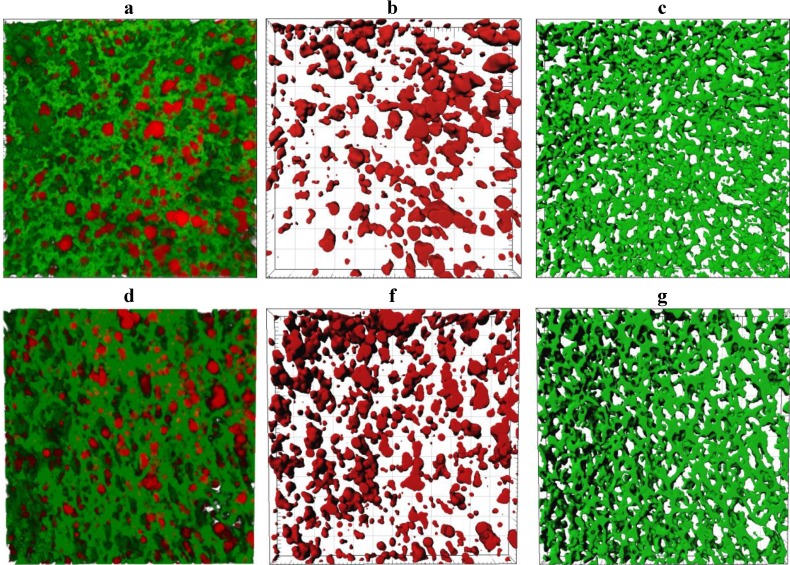
CLSM microstructure of cooked curd made using unconcentrated cheese-milk with ~3.7% w/w milk protein (**a**–**c**) or cheese-milk standardized with LCUFR to 5.8% w/w milk protein (**d**–**f**). All images are 3D reconstructions consisting of 40 layers where the separation between layers is 0.25 µm giving a total observation depth of 10 µm. Nile red stained fat appears red and fast green stained protein appears green. Images **b**–**f** are the rendered volume of the CLSM images, where b and e show the rendered volume of the fat; **c** and **f** show the rendered volume of the protein. All scale bars are 20 µm in length.

Representative CLSM images showing the microstructure of the cooked curd produced at a pilot scale are shown in Figure 6. The number of fat globules decreased significantly (*p <* 0.05) after cooking, possibly due to the coalescence of fat globules (supplementary Figure S2a). The sphericity of the fat globules was reduced in some cases (supplementary Figure S2b) and a concurrent increase in the size of the fat globules was observed (supplementary Figure S2c,d). The porosity of the cooked curd samples was not significantly different (*p* > 0.05) for different treatments but the protein strands within the cooked curd made with ~3.7% w/w milk protein (Figure 6c) were qualitatively thinner and less dense than strands observed within cooked curd made with 5.8% w/w milk protein (Figure 6f).

**Figure 7:**
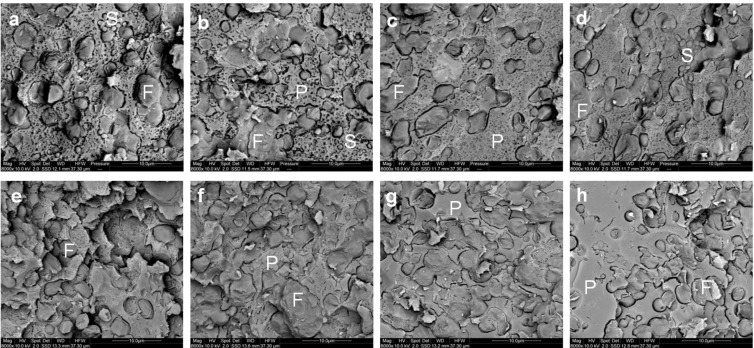
Cryo SEM micrographs of the milled curd (**a**–**d**) and Cheddar cheese (**e**–**h**) made using (**a**,**e**) unconcentrated cheese-milk with ~3.7% w/w protein or cheese-milk standardized with LCUFR to (**b**,**f**) 4% w/w, (**c**,**g**) 4.8% w/w or (**d**,**h**) 5.8% w/w protein, where F = fat, P = protein network and S = starter culture bacteria. Scale bars within the images are 10 μm in length.

The microstructure of the curd after cheddaring and milling at a pilot scale observed by cryo SEM is shown in Figure 7a–d. Micro-pores of about 1 µm diameter could readily be observed within the milled curd made with a lower concentration of milk protein (Figure 7a,b). This was consistent with the higher moisture content of the lower protein cheeses (Figure 3b). Conversely smaller micro-pores of diameter <1 µm were observed within the milled curd made from a higher concentration of milk protein (Figure 7c,d). The starter culture bacteria could also be observed randomly within the curds formed in all treatments.

The micro-pores seen by cryo SEM (Figure 7) could not readily be observed within the CLSM images and fewer differences between the protein treatments in the milled curd microstructure could be observed by CLSM, as also reflected in the data obtained by image analysis for these samples (supplementary Figures S1 and S2).

The microstructure of the cheese after pressing is shown in Figure 7e–h. Pressing completely closes the micro-pores observed earlier within the milled curd (Figure 7a–d). Qualitatively, the microstructure observed by cryo SEM or CLSM for cheese made using the unconcentrated cheese-milk and cheese-milk with 5.8 % w/w protein were not different (Figure 7e–h and supplementary Figure S1). The size of the fat globules within cheeses made from cheese-milk with 5.8% w/w protein, however, was significantly bigger than in cheese made using unconcentrated milk (supplementary Figure S2c,d). This difference may influence the flavour perception and texture of the cheese. 

### 3.6. Texture Profile Analysis of the Cheese

**Figure 8:**
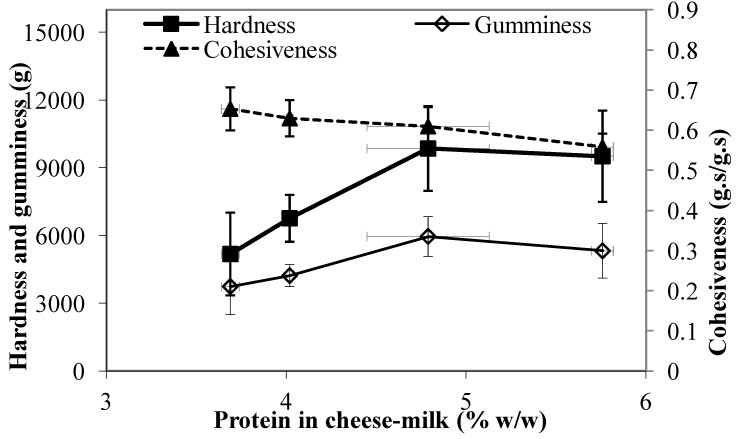
Texture profile analysis showing the hardness, gumminess, and cohesiveness of Cheddar cheeses made using unconcentrated cheese-milk with ~3.7% w/w milk protein or cheese-milk standardized with LCUFR to 4% w/w, 4.8% w/w or 5.8% w/w milk protein. Data are presented as mean ± standard deviation of mean (*n* = 3).

The textural profile of the cheese measured within a week of cheese manufacture is shown in Figure 8. The resistance to compression also known as the hardness, increased as the milk protein level increased (*p* < 0.05) (Figure 8). This result indicates that the cheese structure was more rigid, possibly due to the higher concentration of protein in the cheese-milk during gelation, the difference in microstructure or the lower moisture content of the cheese made using milk with greater protein. No significant measured differences in gumminess were observed (Figure 8). Interestingly, the cohesiveness for cheese made from 5.8% w/w milk protein was significantly lower (*p* < 0.05) than for cheese made from unconcentrated milk (Figure 8). Further, we observed that cheeses made with 5.8% w/w milk protein crumbled more easily during the compression test. 

### 3.7. The Starter Bacteria and Total Lactobacilli Count in the Cheese

Increasing the concentration of starter culture bacteria as the concentration of milk protein increased (Table 1) resulted in a similar population of bacteria in all cheeses (supplementary Table S1). Any variations observed in the microstructural or textural properties of the samples were therefore not due to a different concentration of starter bacteria. The number of the total lactobacilli present was variable between treatments, possibly reflecting variation that can be encountered at a pilot scale in an industrial setting.

## 4. Conclusions

This pilot scale study highlights the potential of using cheese-milk with increasing concentrations of protein to increase the total cheese yield of Cheddar cheese by up to 6% w/w and yield in dry matter by up to 4% w/w for every kg of cheese-milk. These modifications result in minimal changes to the cheese composition, provided the setting time and starter concentration are adjusted during cheese making.

During manufacture, the increased protein concentration decreased the volume of the sweet and salty whey generated, potentially reducing the cost associated with processing this by-product. Less fat was also lost in the salty whey for 5.8% w/w protein samples. Protein addition could also be used as a tool when cheese with a lower moisture content is required and led to only subtle changes in the microstructure, with denser gels, denser milled curds and larger fat globules in the pressed cheese made with milk with 5.8% w/w protein. The cheeses made with higher milk protein (4.8% w/w or 5.8% w/w) were harder than cheeses made with unstandardised milk but not significantly harder than cheeses made with 4% w/w milk protein, which is often used for cheese manufacture. Cheeses made with milk with 5.8% w/w protein were less cohesive, however, suggesting that the optimal concentration may be 4.8% w/w milk protein, where the cheese fat retention was improved by ~7.5% w/w on the basis of the fat levels in the cheese-milk and the cheese yield increased without associated changes in cohesiveness. 

Our results show that structural differences were most significant at the early stage of cheese-making. The bigger pores observed within the cooked curd from the unconcentrated milk may contribute to the higher fat loss observed for these samples and confirms that the practice of standardising milk protein has a positive effect on cheese production. At 5.8% w/w protein concentration, the fat globules within the gel and curd tended to pool together, which possibly contributed to the higher fat loss in the sweet whey. The ability to observe the microstructure of the fat within the curd and cheese using CLSM and cryo SEM gives insights into how fat may be lost during processing. This knowledge may help manufacturers to further optimize the cheese-making process. Our findings also highlight how LCUFR can be added to cheese milk as a tool to improve cheese production and to potentially generate new cheese varieties with different textural characteristics. 

## Supplementary Materials

Supplementary File 1
