# Supplementary material for: Microstructure and Composition of Full Fat Cheddar Cheese Made with Ultrafiltered Milk Retentate
Source: Foods. 2013 Jul 18;2(3):310–31. doi: 10.3390/foods2030310 (PMC5302296; doi:10.3390/foods2030310)
Supplement: Supplementary File 1 [file foods-02-00310-s001.docx]

**Supplementary Information**

**Table S1.** Population of starter bacteria and total lactobacilli in Cheddar cheese made
at pilot scale using cheese-milk with different protein concentrations and matured for
one week ^#^.

| **Target milk protein**  **(% w/w)** | **Viable count (Log CFU/g)** | |
| --- | --- | --- |
|  | **Starter bacteria** | **Total Lactobacilli** |
| 3.5 | 9.83 ± 0.09 | 7.94 ± 0.07 |
| 4.0 | 9.49 ± 0.15 | 7.48 ± 0.39 |
| 5.0 | 9.41 ± 0.17 | 6.67 ± 0.59 |
| 6.0 | 9.39 ± 0.24 | 7.71 ± 1.52 |

^#^ Results are expressed as mean ± standard deviation of mean (*n* = 3); Means across a single column without superscripts are not significantly different (*p* > 0.05); CFU= colony forming units.

**Figure S1.** The 2D CLSM microstructure of samples made using cheese-milk with
a protein concentration of (**a**) ~3.7% w/w, (**b**) 4% w/w, (**c**) 4.8% w/w or (**d**) 5.8% w/w. Images **a**1–**d**1 are from gel samples; **a**2–**d**2 are from cooked curds; **a**3–**d**3 are from milled curds; and **a**4–**d**4 are from Cheddar cheese samples. The Nile red stained fat appears red and the fast green FCF stained protein appears green in these images. All scale bars are
10 μm in length.


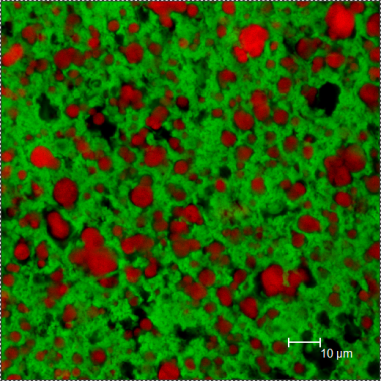

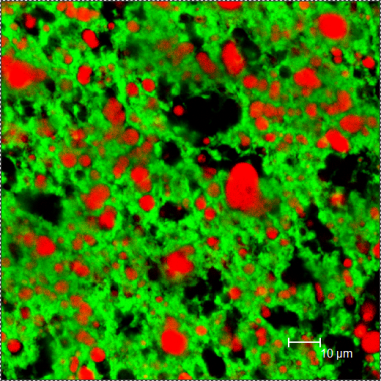

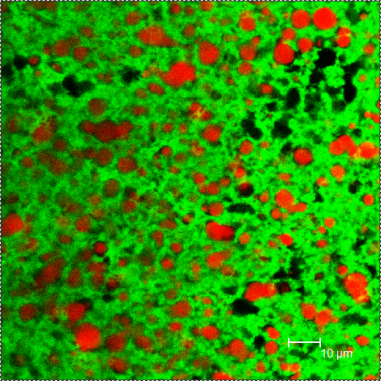

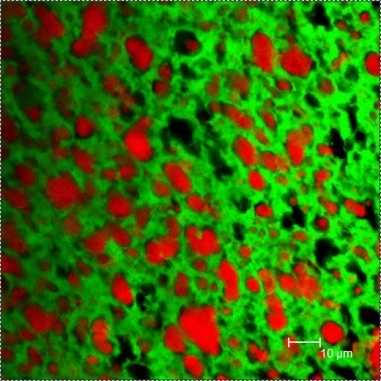

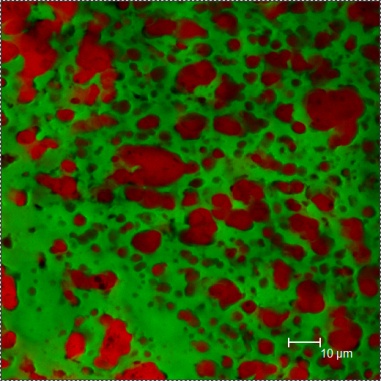

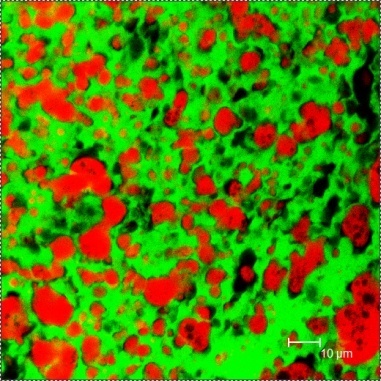

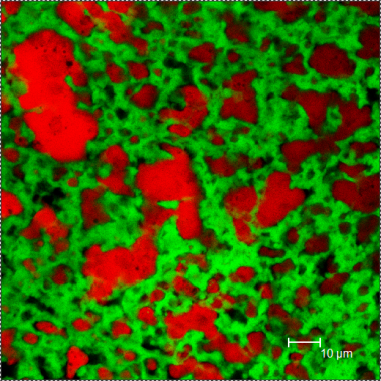

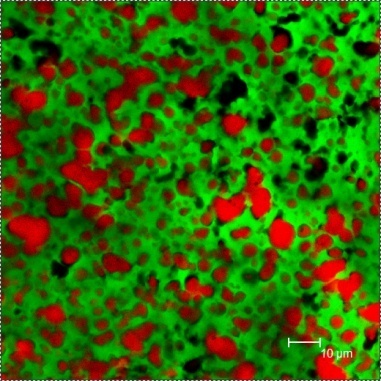

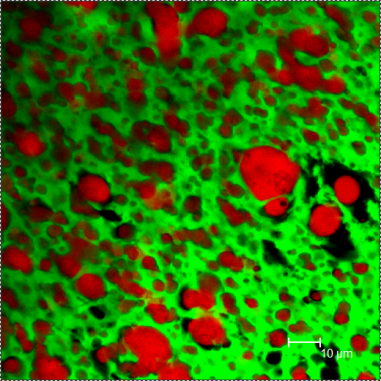

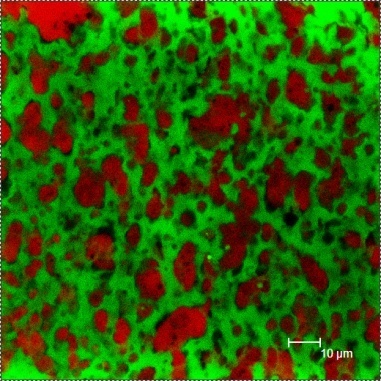

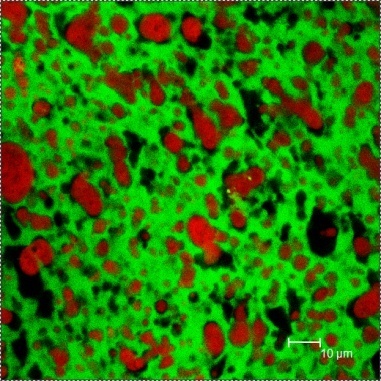

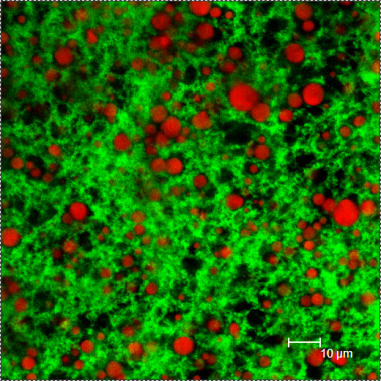

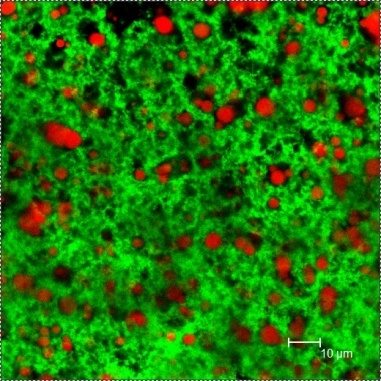

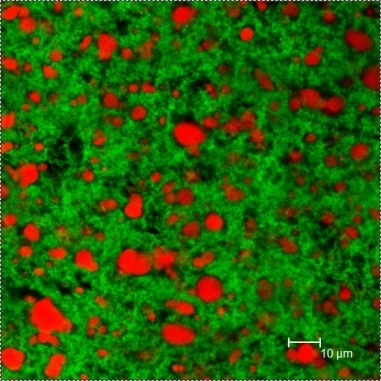

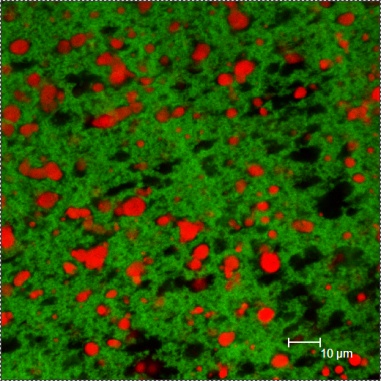

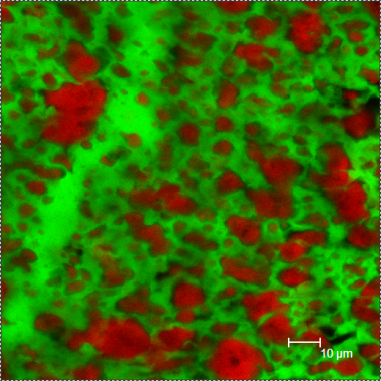


**a1**

**b1**

**c1**

**d1**

**a2**

**b2**

**c2**

**d2**

**a3**

**b3**

**c3**

**d3**

**a4**

**b4**

**c4**

**d4**

**Figure S2.** Physical properties of the gel, cooked curd, milled curd and cheese samples prepared using cheese-milk with ~3.7% w/w, 4% w/w, 4.8% w/w or 5.8% w/w milk protein. All properties were determined by three dimensional image analysis of CLSM images. (**a**) number of fat globules, (**b**) sphericity of fat, (**c**) mean volume of fat, (**d**) fat globule diameter, (**e**) total volume of fat and (**f**) porosity (fraction of pore volume with respect to the total sample volume). Results are presented as mean ± standard deviation of mean (*n* = 6).

|  | |  |  |
| --- | --- | --- | --- |
|  | |  | |

**Figure S2.** *Cont.*

|  |  |
| --- | --- |
